# The prevalence of hepatitis B virus markers in a cohort of students in Bangui, Central African Republic

**DOI:** 10.1186/1471-2334-10-226

**Published:** 2010-07-29

**Authors:** Narcisse P Komas, Souleyman Baï-Sepou, Alexandre Manirakiza, Josiane Léal, Aubin Béré, Alain Le Faou

**Affiliations:** 1 Viral Hepatitis Laboratory, Institut Pasteur de Bangui, PO Box 923 Bangui, Central African Republic; 2 New Affiliation address: Laboratoire de Virologie, CHU de Nancy, Hôpital de Brabois-adultes, 54511 Vandoeuvre-lès-Nancy, France

## Abstract

**Background:**

Hepatitis B virus (HBV) is the major cause of chronic hepatitis, cirrhosis, and hepatocellular carcinoma. The global epidemiological scenario of HBV infection has been changing rapidly over the last two decades due to an effective immunization programme initiated by the World Health Organization. The objective of this study is to estimate the prevalence of HBV in apparently healthy young people and to identify the risk factors of transmission of the HBV among this population in Bangui.

**Methods:**

Dried blood Spots from 801 adolescent high school and young adult university students were prepared by spotting a drop of whole blood (4 spots) from the same fingerprick onto Whatman filter paper. A blood sample aliquot eluted from DBS was then processed with commercial ELISA tests (Abbott Murex, Dartfort, UK) to detect HBsAg antigen, Anti-HBc and Anti-HBs antibodies).

**Results:**

The overall prevalence was 42.3% for antibody to hepatitis B core antigen, 15.5% for HBsAg of which 1.3% of HBsAg alone. HBV familial antecedents, sexual activity and socioeconomic conditions were the main risk factors of HBV infection encountered in the adolescents and young adults.

**Conclusion:**

These results show for the first time the high prevalence of HBV in apparently healthy young people in Bangui. This high prevalence is age- and sex-independent. Transmission risk factors were a familial antecedent of HBV, no utilisation of condoms and public scholarship. To lower HBV prevalence, an adequate program of active screening and vaccination for adolescents and young adults should be implemented, along with a universal immunization program.

## Background

Hepatitis B virus (HBV) infection represents a major health problem, with 2 billion people infected worldwide and more than 400 million chronic carriers of HBV. Globally it causes about 1.2 million deaths per year due to various complications including chronic hepatitis, cirrhosis, and liver cancer [1-4]. It is estimated that about one third of the infected individuals have symptoms and/or biological evidence of hepatitis. It is well established that the earlier the contamination, the higher the risk of chronic infection which is as high as 90% in infected infants. Chronic carriers have a high mortality rate due to complications. In Sub-Saharan Africa, the prevalence of HBV surface antigen (HBsAg) is 3 - 20% and markers of past exposure ranging from 60 - 99% [5].

The Central African Republic (CAR), located in tropical Africa, is considered an area of high endemicity for HBV infection with most HBV strains that belong to the West African genotype E [6]. The transmission of this disease is believed to be mainly by sexual, vertical and intrafamilial routes [7]. Previous study on young sexually active adults, examined in a Public health clinic for sexually transmitted disease in CAR, has shown a high prevalence of HBsAg (14%) with a prevalence of anti-HBc antibodies at 89% [8]. In addition, a survey carried out in the pediatric hospital of Bangui revealed a prevalence of 22.3% in children under 16 years of age with a precocity of the infection (25% of the children were infected before they reached one year) and a high increase of prevalence (48%) among children aged of 10 to 15 [9]. Since these studies have been carried out in the hospital environment they do not obligatorily reflect the prevalence of HBV in the general population. During adolescence, individuals are more prone to risk behaviors such as alcohol abuse, multiple partners and are exposed to sexually transmitted diseases [10,11]. Hepatitis B is a preventable disease and it is necessary to establish its importance and distribution among the population in order to evaluate the need for implementing preventive action [12,13]. Although, CAR has a high prevalence of HBV infections, there is for the moment neither a national program of prevention nor national recommendations to limit the transmission of this disease. Thus, the purpose of this survey was to determine the prevalence of HBV in an apparently healthy population and identify the risk factors of infection among adolescents and young adults in order to propose strategies of prevention.

## Methods

### Study population

The study was carried out from 1^st ^to 28^th ^of February, 2007 in seven (7) public and five (5) private high schools, randomly selected, and in the University of Bangui. The objectives of the survey were presented to each high school Director and to the General Secretary of the University in order to obtain their authorization.

To evaluate the prevalence and risk factors for HBV infection, the study sample was calculated using the Schwartz formula on the basis of an alpha error of 5%, an expected HBsAg prevalence of 22.3% [9] and a precision of 3%. In accordance with these data, the required sample size was 740 individuals.

A total of 801 individuals were sampled and completed the questionnaire. In addition to the standard questions on socio-economic status, the following risk factors were investigated: HBV familial antecedents (at least one of parents or siblings was a chronic HBV carrier), tattooing, body piercing, alcohol and illicit drug consumption, number of sexual partners, age of first sexual intercourse. Individuals were assured that the obtained information would not be shared with parents (except in the case of students under 18 years of age), school staff and any other person. The students were almost evenly distributed among the 8 districts of the city and the adjacent town of Bimbo.

### Ethical approval

Informed consent was obtained from all individuals. For subjects under 18, parental consent was required. Each participant and/or parent was informed of the results of the serology. The study was approved by the Scientific Committee of the Faculty of Medicine of the University of Bangui.

### Specimen Collection

Dried blood spots (DBS) were prepared by spotting a drop of whole blood (4 spots) from one finger prick onto Whatman filter paper. Blood collections were carried out in the amphitheatres and/or the classrooms. Once air-dried, the filter papers were sealed in plastic bags, brought to the laboratory and stored at - 20°C in the presence of a desiccant until testing.

### Serological assays

In this study, we slightly modified the technique of DBS that was previously described [14]. Briefly, blood samples were extracted from DBS by punching one bloodstained circle of 6 mm diameter from the filter paper, and then incubated overnight in 500 μl PBS at room temperature. After a rapid centrifugation, the supernatant was used for testing. HBs antigen Version 3, Anti-HBc and Anti-HBs antibodies (Abbott-Murex Biotech Ltd, Dartford, Kent, UK) were performed according to manufacturer's recommendations on all samples. The value of the technique was ascertained by comparing the results obtained with sera and filter paper eluate for 15 individuals. All HBsAg positive results were confirmed (Murex HBsAg Confirmatory test, Version 3). The interpretation of the serological assays was as stated by the manufacturers.

### Statistical analysis

Epi-Info Version 2000 (CDC, USA & WHO Geneva, Switzerland) software was used for analyzing data: prevalence, 95% confidence intervals and Chi-square test for the comparison of variables and association of HBV positivity and risk factors. Statistical significance was assessed at *p *< 0.05.

## Results

The surveyed population of adolescents and young adults consisted of 455 (56.7%) males and 347 (43.3%) females (sex ratio = 1.31). Age ranged from 14 to 48 years (mean 20.8, SD ± 4.1). They were from public (336, 41.9%) and private (239, 29.8%) high schools and the University of Bangui (226, 28.2%).

The preliminary tests of 15 samples from DBS have shown a good correlation of results with the test performed on sera but for one patient. However, confirmatory test showed that it was a false HBs positivity (table 1). Among the young adults, 339 were HBV positive (Table 2), and of these 124 (15.5%) had an active infection. Ten (10, 1.3%) had "HBsAg only". Prior infections were detected in 215 individuals (26.8%) of whom only 8 (1.0%) had anti-HBs. None of the 801 individuals was vaccinated against HBV.

**Table 1:** Comparison of the sensitivity and specificity of HBsAg and Anti-HBc Ab detection in DBS and sera

		Positive		Negative		Confirmatory test	
		
	n	Sera	DBS	Sera	DBS	Sera	DBS
HBsAg	15	9	10	6	5	9	9
Anti-HBc Ab	15	10	10	5	5	NA*	NA
Anti-HBs Ab	5	1	1	4	4	NA	NA
Sensitivity	HBsAg	100%					
	Anti-HBc Ab	100%					
Specificity	HBsAg	83.3%^#^					
	Anti-HBc Ab	100%					

*NA: Not Applicable

^#^100% after confirmatory test.

**Table 2:** Prevalence of hepatitis B markers detected in 801 students in Bangui

Serological markers present		Number (% of total*)	95% CI
HBsAg		124 (15.5)	13.0 - 18.0
	HBsAg only	10 (1.3)	0.5 - 2.0
	HBsAg, Anti-HBc Ab	114 (14.2)	11.8 - 16.6
Anti-HBc Ab only		207 (25.8)	23.1 - 29.3
HBsAg and/or Anti-HBc Ab		331 (41.3)	37.9 - 44.7
Anti-HBs Ab/Anti-HBc Ab		8 (1.0)	1.2 - 6.2

*801 individuals

There was no statistical difference between the prevalence of HBV infection markers according to sex, age (Table 3) or place of residence. However, students from district 4 which is characterized by a large population living in close proximity had the highest prevalence of HBV infections while those from Districts 2 and 3 with a higher living standard had the lowest.

**Table 3:** HBV positivity according to sex and age among 801 students in Bangui

	Male			Female		
**Age (Years)**	**Total**	*HBV+ (%)	**95% CI**	**Total**	*HBV+ (%)	**95% CI**

14-19	149	41.6	33.6-49.6	195	43.1	36.1-50.1
20-25	234	43.2	36.8-49.6	125	48.0	39.2-56.8
≥ 26	71	32.4	21.2-43.6	27	33.3	14.7-51.9
Total	454	40.1	35.5-44.7	347	45.2	39.9-50.5

*HBV(+): one of the markers (HBsAg, anti-HBc Ab and anti-HBs Ab) at least is positive

Among the risk factors (Table 4), there was no association between HBV infection and socio-economic conditions. However, the prevalence of HBV infection markers was significantly higher: i) in orphans (OR 1.47, 95% CI 1.05-2.06), ii) in individuals attending public high school in comparison to those from private high school or the university (OR 2.8, 95% CI 1.95-4.03; OR 2.38, 95% CI 1.66-3.43), iii) when HBV familial antecedent are reported (OR 1.80, 95% CI 1.21-2.93) and iv) finally, in individuals who did not use condoms (OR 1.69, 95% CI 1.17-2.43) and have 2 or more sexual partner (OR1.87, 95% CI 1.15-3.03). Other variables that may be considered as high risk factors such as body piercing, tattooing, marital status, profession of parents, alcohol use, years since first sexual intercourse, surgery, dental surgery or blood transfusion were associated to a moderate higher risk of HBV infection without any statistical significance. Finally, it has to be noted that three of the eight people who had admitted the use of an illicit drug were infected.

**Table 4:** Risk factors of HBV in 801 students in Bangui (univariate analysis)

Variable	n	%HBV+	Odds Ratio	95% CI
**Parents**				
One or two are dead	196	49.5	1.47	1.05-2.06
Both are alive	605	40	1	
**Education**				
Public High School	336	55.7	1	
Private High School	239	31	2.80	1.95-4.03
University	226	34.5	2.38	1.66-3.43
**HBV Familial antecedent**				
Yes	100	56	1.80	1.21-2.93
No	701	40.4	1	
**Marital Status**				
Single	701	42.4	0.99	0.63-1.64
Married	100	42	1	
**Tattooing**				
Yes	27	42.4	1.00	0.43-2.83
No	774	40.7	1	
**Dental Surgery**				
Yes	185	38.4	1.24	0.87-1.76
No	616	43.5	1	
**Surgery**				
Yes	76	32.9	0.16	0.11-0.22
No	725	43.3	1	
**Body piercing**				
Yes	564	40.6	1.27	0.92-1.74
No	237	46.4	1	
**Blood Transfusion**				
Yes	33	39.4	1.13	0.53-2.45
No	768	42.4	1	
**Alcohol Use**				
Yes	372	43.5	0.91	0.68-1.22
No	429	41.3	1	
**Injecting Drug Use**				
Yes	8	37.5	NA	-
No	793	42.4		
**Use of Condoms**				
Yes	454	39.6	1	
No	177	52.5	1.69	1.17-2.43
**Sexual experience**				
Yes	631	43.7	1.76	0.53-1.09
No	170	37.1	1	
**Years since first intercourse**				
≤ 1	126	44.4	1	
2 - 3	177	48	0.87	0.53-1.41
> 3	321	40.5	1.18	0.76-1.82
**Number of sexual Partner**				
**One (1) partner**				
(Male)	94	47.9	1.02	0.58-1.81
(Female)	126	48.4	1	
**Two (2) or more partner**				
(Male)	312	38.2	1,87	1.15-3.03
(Female)	99	53.3	1	

*NA = Not Applicable because number (n) is very low

## Discussion

In our study, serology was the method of choice as it permits a direct evaluation of the prevalence of HBV infections. The dried blood spot technique gives reliable results with sensitivity and specificity almost identical to serum testing. The collection of blood samples is made easy using finger pricks [15-17]. It reduces the cost of the survey and, as it is almost painless, the risk of refusal is minimized (and there was none in this study). The high prevalence of HBV infection in young individuals from Bangui, with global HBsAg prevalence of 15.5% clearly confirms the complete absence of any program to combat hepatitis B. This is evident when comparing the rates in developed countries [18,19], or countries who have implemented prophylaxis of this infection [20-22]. The vaccination of infants in the first 11 months will have only a limited impact unless a follow up is implemented and the prevention of mother to infant transmission put in place.

The population of adolescents and young adults in our study consist of all 8 districts of Bangui and from the adjacent city of Bimbo. To determine the risk factors of transmission of HBV in the chosen population, it would be better to survey separately each social level of the population, but, in the absence of any HBV education program in the country it would be difficult to reach the entire population. Public high schools in Bangui and the University are attended by student whatever their social status and the place they live in. However, private schools accept solely students who can afford the fees and are thus from middle and upper class. They represent at least 30% of the adolescent and young adult population. Thus, our study population represents the educated adolescents and young adults living in an urban environment. This population is selected and thus do not reflect the general population. A survey should be conducted with the help of the health authorities to evaluate the rate of HBV infection in the general population.

The prevalence of HBsAg in our study was not correlated with age. As indicated by the survey in children [9] most chronic infections in Bangui are related to mother to infant transmission or infection at a young age. Acquired infection, mainly sexually, in later age, resolves for it most part spontaneously. Thus the prevalence of anti-HBc increases regularly while the one of HBsAg augments only moderately.

Only 3.7% of participants have detectable anti-HBs antibodies in this study. This observation requires further investigation to determine the reason why this study shows such a very low detectable anti-HBs antibody level. This is not due to a loss of the sensitivity of the DBS as it had been shown with the preliminary tests as well as observations from other studies [14,17]. Although infection in neonates can explain an immunotolerance phenomenon, the absence of anti-HBs in young adults is puzzling and requires further investigation. This may be due to some characteristics of the population. A variant of the HBs-antigen may be readily detected while the corresponding antibodies are not. It remains to be determined if this observation is related to the E genotype [6,23].

In our study, there was no evident direct association between socio-economic conditions and HBV infections. However, although not significant, district with low income correspond to higher proportion of infected individuals. Thus it is likely that geographic origin and income are two dependant variables. Nevertheless the role of socio-economic status is reinforced by the higher prevalence of infection in students from public high schools in which they are accepted free of charge. This is not the case in private schools, which only part of the population can afford to attend, as already observed [24]. The same conclusion can be made for the students who attend the university.

In this study, HBV infection may also be mainly linked to sexual transmission. In fact, risk factors, such as lifetime number of sexual partners > 2 and non use of condoms, were associated with HBV in the univariate analysis. This is not surprising as sexual transmission is an important route of HBV infection. The role of drug use cannot be estimated as only eight students declared use of injecting-drugs of whom three were infected with HBV. Intrafamilial transmission may also play a role in HBV infection of young people as living in a family in which one member is infected is also a risk factor [25,26]. Dental care is a moderate risk factor, likely because of lack of hygiene. Contrarily, hospitalization seems to have no role in HBV transmission.

## Conclusion

This survey, the first performed in a population of young healthy educated adults in Bangui, indicates a high prevalence of HBV infection. The increase in anti-HBc prevalence in teens as previously described [9] and the one we observed are in favor of sexually acquired infection. However the environment plays an important role as the absence of education and the low income favor HBV infection. Altogether, the high prevalence of infected young adults reflects the absence of health policy for fighting against this disease. This is alarming and taking into account the high risk of HBV transmission in the country by health authorities may reduce dramatically the burden of this disease. Information of the population, focusing mainly on adolescents, vaccination not only of infants but also of the entire population and a prophylaxis of mother to infant transmission are to be implemented.

## List of abbreviation

Anti-HBc Ab: anti-hepatitis B core antibody; Anti-HBs Ab: anti-hepatitis B surface antibody; Anti-HDV antibody: Anti hepatitis Delta virus antibody; CAR: Central Africa Republic; CDC: Center for Diseases Control; DBS: Dried blood spots; HBsAg: Hepatitis B surface antigen; HBV: Hepatitis B virus; PBS: phosphate buffer saline; SD: standard deviation; WHO: World Health Organization

## Competing interests

None of the authors in this study has any financial personal relationship with any organization that could influence (bias) this work.

## Authors' contributions

NPK designed the study and directed its implementation, supervised all the field activities, analyzed and interpreted data and redacted the manuscript. ABS helped to supervise the field activities, contributed to the acquisition of data by preparing the questionnaire and by participating to the collect of DBS. AM contributed to the analysis and interpretation of the data. JL and AB contributed to the acquisition of data by performing ELISA test and participated to the collection of the DBS. ALF contributed to the analysis and interpretation of the data and to the redaction of the manuscript.

All authors approved the final version of the manuscript.

## Pre-publication history

The pre-publication history for this paper can be accessed here:

http://www.biomedcentral.com/1471-2334/10/226/prepub
